# Educating and Informing Patients Receiving Psychopharmacological Medications: Are Family Physicians in Pakistan up to the Task?

**DOI:** 10.1371/journal.pone.0004620

**Published:** 2009-02-27

**Authors:** Hammad Ashraf Ganatra, Hadi Bhurgri, Roomasa Channa, Fauzia Ahmad Bawany, Syed Nabeel Zafar, Rafia Ishfaq Chaudhry, Syeda Hina Batool, Abdul Basit, Mehmood Asghar, Sarah Saleem, Haider Naqvi

**Affiliations:** 1 Medical College, The Aga Khan University, Karachi, Pakistan; 2 Department of Community Health Sciences, The Aga Khan University, Karachi, Pakistan; 3 Department of Psychiatry, The Aga Khan University, Karachi, Pakistan; University of Bern, Switzerland

## Abstract

**Introduction:**

Studies have shown a high prevalence of psychiatric illnesses among patients in primary health care settings. Family physicians have a fundamental role in managing psychiatric illness with psychopharmacological medications. Providing information about the disease, its management and the potential adverse effects of the medications is an important part of the management of mental illnesses. Our objective was to determine if patients who were prescribed psychopharmacological drugs by family physicians at a community health center in Karachi, Pakistan were provided adequate education about their disease and its management.

**Methods:**

A cross-sectional study was conducted at the Community Health Centre (CHC), Aga Khan University Hospital Karachi, Pakistan. Details about the prescriptions and patient education were acquired from the patients after their consultations.

**Results:**

A total of 354 adult patients were interviewed during 3 days. Among them, 73 (20.6%) were prescribed psychopharmacological medications. Among patients receiving psychopharmacological medicines, 37 (50.7%) did not know their diagnosis; 50 (68.5%) were unaware of the disease process; 52 (71.2%) were unaware of alternative treatments; 63 (86.3%) were not cautioned about the potential adverse effects of the drugs; 24 (32.9%) were unaware of the duration of treatment and in 60 (82.2%) of the participants an appropriate referral had not been discussed. For all aspects of education, patients prescribed psychopharmacological medications knew less as compared to those patients that were prescribed other medications.

**Discussion:**

The practice of imparting information to patients who receive psychopharmacological medications seems to be inadequate in Pakistan. We have hypothesized about the possible reasons for our findings, and identified a need for further research to determine the cause for such findings and to address them accordingly. At the same time there is a need to educate family physicians in Pakistan about the special importance of providing adequate information to such patients.

## Introduction

Common psychiatric illnesses like anxiety and depressive disorders are highly prevalent all over the world. Estimates from 2001 ranks depression among the top ten causes of disability worldwide[1]. Depression is projected to become the second leading cause of disability in the world while the foremost cause in the underdeveloped countries by the year 2020[2]. The mounting burden of such disorders presents more of a challenge for the developing world, where only a minor percentage of the gross domestic product is allocated to health care.

The role of family physicians is fundamental in managing common psychiatric illnesses. Several studies have shown that the prevalence of psychiatric illnesses among patients visiting primary health care providers is high. The prevalence of psychiatric morbidity in Pakistan ranged from 10% to 25% for men and 25% to 57% for women in an analysis of 20 community based studies[3]. Sabooh et al reported the prevalence of common psychiatric illnesses in primary care settings to vary from 17% to 30%[4].

The prescription of psychopharmacological medications plays a major role in the management of common mental disorders. In Pakistan, there are currently only 350 psychiatrists for a population of 150 million[5]. The high prevalence of psychiatric illnesses in primary care clinics often leads to hasty diagnostic and therapeutic decisions. Evaluation and diagnosis of common mental disorders like anxiety and depression involves a challenge to the clinical ability, knowledge and experience of the family physicians. Due to the dearth of psychiatrists, most patients with psychiatric illnesses are being managed by family physicians[6]. Therefore it is imperative to know how they fare when it comes to prescribing psychopharmacological medications and if adequate information about the disease is being provided. Providing adequate information about the disease and its management is of special importance in the treatment of patients with mental illnesses. There is evidence that adequate information helps improve patient cooperation, medication compliance, tolerance to unwanted effects and the efficacy of medications and reduce the rate of unwanted drug effects[7].

The purpose of our study was to determine if patients who were prescribed psychopharmacological drugs by family physicians at a reputable community health center in Karachi, Pakistan receive adequate education about their disease and its management.

## Methods

We carried out a cross-sectional study among patients attending the family medicine clinics at the Community Health Centre (CHC) at the Aga Khan University Hospital (AKUH), Karachi. Karachi is the largest city of Pakistan with an estimated population of 18 million[8]. AKUH is a privately managed tertiary care, university hospital, located in the centre of the city. The CHC provides primary health care services within AKUH premises and it serves patients from all socio-economic backgrounds. It comprises of 10 clinics which are conducted by trained family physicians that are certified by local or foreign examination boards. According to current estimates, the CHC has an average daily turnover of 250 patients. This number includes adults and children attending family medicine clinics, as well as specialty clinics and those coming in for brief surgical and medical procedures.

### Sampling Method

We interviewed all adult patients attending the family medicine clinics at the CHC over a period of 3 days in January 2008. They were consecutively approached for an interview after their consultation was over and they had received their prescriptions. Patients attending specialty clinics, or those who were less than 18 years of age were excluded from the study.

### Questionnaire and Data Collection

We designed a structured interview based questionnaire which comprised of 3 parts. The first dealt with the socio-demographic profile of the participants (e.g. age, sex, education, marital status). The second part documented the drugs prescribed during the consultation. The third and final part assessed whether the patients had been provided adequate information about their diagnosis and its prognosis and about the drugs that they had been prescribed (e.g. adverse effects, duration of treatment)

We originally designed the questionnaire in English and then translated it into Urdu. To ensure that the original wordings were preserved, it was then back translated into English by three different non-medical individuals not related to the study. We then pre-tested this final Urdu questionnaire on 36 subjects to identify any shortcomings of the questionnaire. Some minor problems were identified and we made the appropriate modifications. Data from the pre-test was not included in the final analysis.

The interviews were conducted by a group of senior medical students who were briefed about the study protocol and trained in survey techniques. The interviewers assembled at the CHC, before the start of the clinics. All patients were approached after their consultation was over, and they had received their prescriptions. After taking informed consent, they were interviewed, and their responses were recorded on the questionnaire. Throughout the period of data collection, the family physicians were unaware of the study going on outside their doors. This was intentional and important for the study design.

During the interview, all participants were asked to show their prescriptions which were then noted down in the questionnaire. Before data entry, all the trade names were converted into generic names using PharmaGuide[9], and all psychopharmacological medications were coded into different classes according to their respective modes of action like anti-depressants, anxiolytics, etc. and their subtypes.

### Statistical Analysis

We performed data entry on Epi Data version 3.1 and statistical analyses were done using Statistical Package for Social Sciences 14.0 (SPSS, Inc., Chicago, IL, USA). Descriptive statistics including means and standard deviation (SD) were calculated for continuous variables, while proportions were calculated and tabulated for categorical variables. Demographic details of patients receiving psychopharmacological drugs or other medications were tabulated. The Pearson chi-square test and Independent T-test were used for categorical and continuous variables respectively, to assess for significant differences between the two patient groups. We used the Fisher's exact test to assess if the patient education differed between those receiving psychopharmacological drugs and those receiving other kinds of prescriptions.

### Ethical Considerations

The study was approved by the Ethical Review Committee of the Department of Community Health Sciences at AKUH. We also obtained permission from the Department of Family Medicine.

An informed consent form in Urdu was also attached with the questionnaire which informed all potential participants about the benefits and harms of the study. The interviewers read out the consent form to participants who were unable to read it. Written informed consent was taken from all participants before proceeding with the interview. The questionnaire was anonymous and guaranteed the confidentiality of the study participants as it did not document any information that could link individuals to the study.

## Results

We approached a total of 368 patients aged 18 and above over a period of 3 days, out of which 354 participated in our study with a response rate of 96.2%. Of the total participants, 251 (70.9%) were married, and 220 (62.1%) were unemployed. There were a total of 230 females which comprised 65.0% of the participants. The mean age of the participants was 38.8 years (SD 15.3). Of the participants, 130 (36.8%) were illiterate or had received primary education only. 54.5% had come in for a follow-up visit and 71.5% were financing their own healthcare. All patients who were interviewed had received a prescription from their family physician, either in the form of therapeutic or symptomatic medication. Prescription of psychopharmacological medications was found among 73 (20.6%) of the participants. **Table 1** presents the differences in demographic profiles of the patients receiving psychotropic medications versus those receiving other medications. Among the patients prescribed psychopharmacological medications, 35 (47.9%) received Selective Serotonin Reuptake Inhibitors (SSRI), 23 (31.5%) received Tricyclic Antidepressants (TCA) and 15 (20.5%) received Benzodiazepines (BDZ).

**Table 1:** Demographic profile of the patients receiving psychopharmacological medications and patients receiving other medications (n = 354).

Variable	Patients receiving psychopharmacological drugs (n = 73) Number (%)	Patients receiving other drugs (n = 281) Number (%)	P-Value*
**Gender**			
Male	13 (17.8)	111 (39.5)	0.001
Female	60 (82.2)	170 (60.5)	
**Age**			
Age Range (Min–Max) in Years	18–79	18–85	0.005§
Mean Age in Years±SD	43.3±15.4	37.6±15.0	
**Marital Status**			
Unmarried	10 (13.7)	85 (30.2)	0.011
Married	61 (83.6)	190 (67.6)	
Widowed	2 (2.7)	2 (0.7)	
Divorced	0 (0)	4 (1.4)	
**Highest level of education**			
Illiterate	21 (28.8)	50 (17.8)	<0.001
Primary	14 (19.2)	45 (16.0)	
Secondary	21 (28.8)	38 (13.5)	
Intermediate	5 (6.8)	32 (11.4)	
College Graduation	10 (13.7)	100 (35.6)	
Post graduation	2 (2.7)	16 (5.7)	
**Employment status**			
Unemployed (*including housewives and job seeking individuals*)	53 (72.6)	167 (59.4)	0.096
Employed	17 (23.3)	103 (36.7)	
Retired	3 (4.1)	11 (3.9)	
**Total monthly household income (in Rupees)**			
<10,000	27 (37.0)	88 (31.3)	0.519
10,000–45,000	35 (47.9)	140 (49.8)	
45,000–100,000	8 (11.0)	46 (16.4)	
>100,000	3 (4.1)	7 (2.5)	

* Pearson Chi-square was used to calculate p-values.

§ Independent Samples T-Test was used to calculate p-value.

For all aspects of education, patients prescribed psychopharmacological medications knew less as compared to those patients that were prescribed other medications. As shown in **Table 2**, among the patients receiving psychopharmacological medications, 50.7% did not know their diagnosis compared to 27.9% of patients with other medications *(p-value<0.001)*; 68.5% (versus 48.4%) of the participants were not explained the disease process in layman terms *(p-value = 0.002)*; 71.2% (versus 56.2%) of the participants were not counseled about alternative treatments *(p-value = 0.020)*; 86.3% (versus 77.7%) of the participants were not explained the adverse effects of the drugs *(p-value = 0.107)*; 32.9% (versus 23.3%) were not aware of the duration of treatment *(p-value = 0.093)* and with 82.2% (versus 74.9%) of the participants an appropriate referral had not been discussed *(p-value = 0.192)*.

**Table 2:** Teaching of patients prescribed psychopharmacological medications vs. those who were prescribed other medications (n = 354).

Variables	Patients receiving psychopharmacological drugs (n = 73) Number (%)	Patients receiving other drugs* (n = 281) Number (%)
**Did the patient know his/her diagnosis?**		
Yes	36 (49.3)	199 (72.1)
No	37 (50.7)	77 (27.9)
	(p value<0.001)	
**Did the patient know about the disease process?**		
Yes	23 (31.5)	142 (51.6)
No	50 (68.5)	133 (48.4)
	(p value = 0.002)	
**Were alternate treatment options discussed?**		
Yes	21 (28.8)	120 (43.8)
No	52 (71.2)	154 (56.2)
	(p value = 0.020)	
**Were adverse effects explained?**		
Yes	10 (13.7)	61 (22.3)
No	63 (86.3)	213 (77.7)
	(p value = 0.107)	
**Did the patient know the approximate duration of treatment?**		
Yes	49 (67.1)	211 (76.7)
No	24 (32.9)	64 (23.3)
	(p value = 0.093)	
**Was an appropriate referral discussed?**		
Yes	13 (17.8)	69 (25.1)
No	60 (82.2)	206 (74.9)
	(p value = 0.192)	

Fisher's Exact Test was used to calculate the p-values.

* Numbers in the column “Other drugs” do not add up due to non-responders among this group who did not divulge particular information about their consultation.

## Discussion

Questions have been raised about the relevance of psychopharmacological prescriptions in primary health care practice[10]. These drugs can have serious side effects and some of them, especially the benzodiazepines have an addictive potential. Therefore, their prescription and use should be monitored by a healthcare professional. Also, the treatment of mental disorders entails more than just prescription of medicines; it also requires the doctor to develop a strong bond with the patient, and to counsel the patients regarding alternative treatment options. In this context, several of our findings were distressing.

Almost 51% of the patients with psychopharmacological medications did not know their diagnosis. Several factors could be responsible for this. Most of the psychopharmacological medicines in our study were antidepressants and it is possible that doctors were hesitant to tell patients that they were suffering from depression. Further, patients may have refused the diagnosis due to the social stigma associated with psychiatric diseases, or they may have been unable to understand the diagnosis due to cognitive disability associated with psychiatric illnesses. At the same time it can also be argued that the doctors were unable to effectively explain the diagnosis to their patients because they themselves were unclear about it.

With almost 71% of the patients no alternative treatment options were discussed. This issue must be addressed because addition of non-pharmacological treatment options can be helpful in augmenting the treatment of common mental disorders like anxiety and depression, and in milder cases these may be the only forms of treatment required[11], [12]. This finding can also be interpreted as a sign of over dependence on medications on the part of family physicians when it comes to managing such disorders, given that often there are several pharmacological and non-pharmacological treatment options.

Adverse effects of the drugs had not been explained to almost 86% of the patients being prescribed psychopharmacological medications. SSRIs have fewer side effects, but TCAs and benzodiazepines have significant ill-effects which should be discussed with the patients before initiating therapy. In general, most patients attending primary care services want to know the adverse effects of the treatment being prescribed to them[13]. Although, most patients knew about the duration of treatment, a third did not. This can prove hazardous as prolonged treatment with benzodiazepines can lead to dependence[14], [15], while stopping antidepressants before time is likely to lead to relapse[16].

With almost 82% of the patients no referral plan was discussed. This complements the findings of a previous study which showed that only 3% of the patients in a psychiatry clinic had been referred by primary care physicians[6]. The low rate of referral makes it all the more important for the physicians at first level of care to be well versed in the management of mental illness. In a developing country like Pakistan mental illnesses make up a significant proportion of the total disease burden and trained psychiatrists are few. Consequently, correct identification and effective management of common psychiatric disorders by family physicians becomes essential[17].

The most common psychopharmacological medicines prescribed were antidepressants with relatively fewer prescriptions of benzodiazepines. This pattern differs from other studies which show benzodiazepines rather than antidepressants as being the most commonly prescribed psychopharmacological medicine[18], [19]. This is an encouraging finding as benzodiazepines have a high potential for addiction and should be used judiciously. The doctors in our study were trained, local or international board certified family physicians who had been exposed to psychiatric patients during their training and were therefore able to make a more informed decision about benzodiazepine prescription. This may explain the low prescription rate of benzodiazepines. It may be worthwhile to carry out a similar study among primary care physicians without specialized training to know how they fare when prescribing psychopharmacological medications. Postgraduate training of family physicians in treating common psychiatric disorders has been suggested to protect patients against unnecessary prescription of psychopharmacological medications [20].

On the whole, all aspects of providing information to patients receiving psychopharmacological prescriptions were inadequate. This is a cause of concern as the doctors involved in our study were trained family physicians, whereas most primary care doctors in Pakistan receive no such training. A recent study showed that although there are very few formal training programs for family medicine in Pakistan, they are of international standards. However, very few doctors go through these training programs and most of them venture into family practice right after their house jobs without any continued training[21]. There is no reason to believe that their performance would be any better, in fact it is our belief that it would be worse. Given that the quality of mental health care provided by untrained family physicians and self-proclaimed medical workers (“quacks” and “hakims”) in Pakistan is not known, there is a need to assess how less specialized health care providers fare when managing patients with suspected psychiatric illnesses.

At the same time, measures are needed to improve the performance of existing trained and untrained family physicians, and to educate them about the importance of providing information to patients receiving psychopharmacological medications. This can be done by enrolling them in Continuous Medical Education seminars or special courses and workshops throughout the country Such interventions have been suggested and proven beneficial in other countries too[22], [23].

However, additional factors might be responsible for the inadequate practice of patient education seen in our sample. As shown in Table 1, the study participants who received psychopharmacological drugs differed from other patients with regards to gender, mean age, marital status and educational level. There were more illiterate or less educated individuals, and less college graduates and postgraduates. This suggests that these patients were adequately educated about their disease by the physicians but had a poor retention or comprehension of the counseling session due to their lower educational status. Similarly, the group of patients receiving psychopharmacological drugs had a higher proportion of females (82.2%) as compared to those with other medications (60.5%). Females in Pakistan generally have a lower educational status, and the lower quality of counseling reported by females might be a reflection of their poor understanding and comprehension secondary to lower education.

The patients with psychopharmacological medication were also older than those receiving other medications (43.3 years vs 37.6 years, *p-value 0.005*). A greater number of elderly patients in one group might have had cognitive defects leading to poor comprehension of the information provided by the physician. Also, the family physicians might have had problems in communicating with the elderly patients.

It is also possible that a quantity driven and profit oriented clinic setting makes it difficult to adequately counsel patients due to practical constraints like lack of time. In a previous study it was speculated that market influences were a possible reason for poor prescribing practices, as physicians in Pakistan participate in a market where consultations including patient education and medications are bought and sold[24]. Such constraints might be overcome to some extent if standardized patient leaflets were used to facilitate communication. Further, it is possible that only “difficult” or chronic patients received psychopharmacological medications due to feelings of helplessness on part of the physician. This might have led to a poorer quality of patient education in these cases as a clear plan for the treatment was not present. It has also been argued that the poor level of mental health literacy among patients could be a reason for the inadequate management of psychiatric illnesses in the country[25]. It was not our objective to pin-point the specific reasons behind this inadequate counseling but to merely determine if it is an issue of concern or not.

There were certain limitations to our study. Our data was collected from 2 sources i.e. the patients' prescriptions and the questionnaire based interviews. As regards the interview, some participants had been diagnosed with common mental disorders like anxiety and depression, or were taking psychopharmacological medications. This might have introduced an element of information bias in our study as both these factors are known to cause cognitive impairment. Also, our study was a cross-sectional survey and we recruited patients consecutively. It is an inherent limitation of the study that we cannot comment on the representativeness of the patients recruited during the three days. While all prescribing CHC doctors were trained and accredited family physicians, this setting cannot be generalized to the general practitioners throughout the country which comprise mostly of untrained doctors. The fact that there were multiple interviewers could also have led to an element of interviewer bias. However, we strived to eliminate this by designing a standard questionnaire and all interviewers read out the same standard set of questions during the interview.

### Conclusion

Pakistan is a developing country with limited healthcare resources and very few mental health professionals. In such a setting, family physicians have a responsibility of effectively managing mental illnesses; effective education of the patients about their disease and treatment options is a vital component of disease management. Our study gives an insight into how family physicians in Pakistan prescribe psychopharmacological drugs and shows that, when it comes to imparting information and education, there are significant communication gaps between physicians and patients. It shows that a significant proportion of patients attending primary care services receive psychopharmacological medication without adequate education about their disease and its management. We have hypothesized about factors that may be responsible for this including physician-related factors like poor training, time constraints and profit motivated practice, and patient-related factors like low education, older age and poor level of mental health literacy among patients. However, the definite reasons for the lack of proper patient education should be explored by further research. At the same time there is a need to educate family physicians in this regard and to develop strategies to improve their communication skills.
